# Entropy decay during grain growth

**DOI:** 10.1038/s41598-020-68569-z

**Published:** 2020-07-17

**Authors:** Pawan Vedanti, Xin Wu, Victor Berdichevsky

**Affiliations:** Department of Mechanical Engineering, Wayne State University, Detroit, MI 48202 USA

**Keywords:** Metals and alloys, Mechanical engineering

## Abstract

Materials with random microstructure are characterized by additional thermodynamic parameters, entropy and temperature of microstructure. It has been argued that there is one more law of thermodynamics: entropy of microstructure decays in isolated systems. In this paper, we check this assertion experimentally for the process of grain growth. We show that entropy of grain structure decays indeed as expected. We study also the equation of state for microstructure entropy. In general, microstructure entropy should be a function of microstructure energy and the average grain size. We observed that the equation of state degenerates, and there is a universal dependence of microstructure entropy on microstructure energy, at least at the stage of self similar grain growth.

## Introduction

It has been recognized in various branches of material science that thermodynamic description of materials with microstructure requires two additional thermodynamic parameters, entropy of microstructure and temperature of microstructure. Such parameters have been mentioned under different names in theory of granular materials^1–3^, metal glasses^4–18^, crystal plasticity^19–24^, composite materials^25^, and grain growth^26–28^. Moreover, it was claimed that there is one more law of thermodynamics:* entropy of microstructure must decay in isolated systems*^29^. One mechanism of this special way of evolution is due to the dissipative nature of mesoscopic dynamics. Dissipative equations possess attractors and trajectories of the system in phase space must fall on the attractor^30^. If entropy of microstructure is associated with the volume of phase space as in classical statistical thermodynamics, then the entropy of microstructure must decay as phase volumes moving to an attractor shrink. A different mechanism of microstructure entropy decay is characteristic for driven dissipative systems such as slow plastic deformation of crystals and polycrystals^31,32^. In this work, we aim to check the entropy decay experimentally. We choose the process of grain growth as the testing ground. Grain growth is ideally fitted to such experimental study, because it can proceed in an isolated setting. This can be seen from the following thought experiment. If a polycrystal is heated enough to allow for grain boundary motion to proceed and then thermally isolated grain growth sets up and does not stop as grain boundary motion heats the crystal. The higher temperature increases grain boundary mobility and the process does not stop. In the actual experiment we employ the isothermal setting assuming that the results are similar. We check the consistency of this assumption in section 4.

There is an ambiguity in the choice of entropy.(Further we call microstructure entropy briefly entropy using for usual entropy the term thermodynamic entropy, as it will appear further in the paper only in Section IV.) of grain boundary structure. The notion of entropy is multifaceted, and the choice depends on the context in which entropy is used. We aim at a macroscopic description of grain growth when the process is described by a few averaged parameters. In classical thermodynamics, entropy arises inevitably as an unavoidable parameter in constitutive equations. Is the situation in grain growth similar? In principle, to answer this question one has to develop an average description of grain structure dynamics. This is a formidable task at the moment. It is enough to mention that, formally speaking, grain boundary is an infinite-dimensional object. Though infinite dimensionality is artificial because grain boundary pieces with sizes that are smaller than the interatomic distance do not carry independent degrees of freedom, and some short wave truncation must be made in grain boundary dynamics, a convincing high-dimensional analysis of grain boundary dynamics does not seem to exist. Besides, grain boundary dynamics is not governed by just mean curvature flow as it is also affected by impurities, number of grain sides and properties of vertices and grain edges^33–35^. This makes the choice of proper finite-dimensional truncation a quite non-elementary issue. Some finite-dimensional models have been discussed in^36–44^. Here we will employ the crudest dynamic model possible: it presents the grain boundary structure as a “gas of grains”, where each grain is characterized by one number, either grain volume or grain radius. Grains can grow and shrink and do not have “energetic” interactions, i.e. the total energy of the grain structure is the sum of energies of individual grains with the factor 1/2 as each piece of grain boundary provides the same contribution to energies of two neighboring grains. The interaction of grains arises from the kinematic constraint: the sum of volumes of all grains is preserved. This model goes back to the work by Hillert^45^, and was further developed in many studies^46–49^. Hillert obtained an equation for probability distribution of grain sizes. This equation was modified by Berdichevsky^27^ to allow for analytical solutions . Analytical solutions made it possible to observe that the expected features of entropy holds, if one means by entropy the usual Boltzmann entropy. We distinguish the total entropy of the grain boundary microstructure ${\mathbb {S}}_{m}$ and entropy per one grain $S_{m}^{*}$,

1 $$\begin{aligned} {\mathbb {S}}_{m}=NS_{m}^{*}, \end{aligned}$$

here index *m* stands for microstructure, *N* being the number of grains. Entropy per one grain $S_{m}^{*}$ is the Boltzmann entropy.

2 $$\begin{aligned} S_{m}^{*}=-\int f(v)\ln (f(v)v_{0})dv, \end{aligned}$$

where *f*(*v*) is the probability distribution of grain volumes, $v_{0}$ some characteristic grain volume.

All parameters in (1) and (2) evolve in the course of grain growth. In the analytical study^27^, parameters $S_{m}^{*}$ and ${\mathbb {S}}_{m}$ change in opposite directions: entropy per grain $S_{m}^{*}$ increases, while total entropy ${\mathbb {S}}_{m}$ decays. Increase in $S_{m}^{*}$ indicates the chaos enhancement while the decay of ${\mathbb {S}}_{m}$ corresponds to the general concept of entropy decay in closed systems. Besides, there is an equation of state: entropy is a function of total energy of grain boundaries $E_{m}$ and average grain volume *v*,

3 $$\begin{aligned} {\mathbb {S}}_{m}\mathbb {={\mathbb {S}}}_{m}\mathbb {(}E_{m},v\mathbb {)}. \end{aligned}$$

In the work reported here, we study the evolution of $S_{m}^{*}$ and ${\mathbb {S}}_{m}$, and the validity of the equation of state (3). Briefly, the results are as follows: total entropy ${\mathbb {S}}_{m}$ decays as expected, entropy per one degree of freedom $S_{m}^{*}$ fluctuates slightly not showing a certain trend, while the equation of state (3) degenerates into equation of the form ${\mathbb {S}}_{m}\mathbb {={\mathbb {S}}} _{m}\mathbb {(}E_{m}\mathbb {)}$.

## Experimental setup

Measuring the evolution of grain boundary structures is a toilsome task, and we replace it with observations of the grain boundary traces on the specimen surface. As-recieved nickel pieces (commercially pure nickel from McMaster–Carr) were annealed and then polished to scan the specimen grain boundaries and orientation by Electron Back Scatter Diffraction (EBSD). The EBSD image was used to get information of individual grain areas and perimeters by hand-tracing the grain boundaries. We measured mean cross-sectional grain area ${\bar{a}}$ and mean cross-sectional perimeter ${\bar{p}}$ independently and studied their evolution in the course of grain growth. We also worked out the reported results for aluminum alloy Al 5083F and magnesium alloy AZ31b Mg obtained by Wu^58^ and Bhattacharya et al.^59^, respectively. For all the three materials used during the grain growth experiments, grain boundary mean cross-sectional area ${\bar{a}}$ increases 100–350 times while mean cross-sectional grain perimeter ${\bar{p}}$ increases by a factor of 15–20. The grain size distribution for almost all samples was very close to self-similar distribution. Further details on the experiments can be found in^60^.

## Results

### Entropy decay

To find entropy from these experiments one has to specify a finite-dimensional version of (2). As such we use the relation,

4 $$\begin{aligned} S_{m}^{*}=-\sum p_{i}\ln p_{i} \end{aligned}$$

Probabilities $p_{i}$ in (4) are interpreted in the following way: the possible values of grain sizes are split in bins and $p_{i}$ is the portion of grains in the i^th^ bin. In such interpretation, the values of $S_{m} ^{*}$ depend on the bin size. To minimize the bin size dependence, we average $S_{m}^{*}$ over various values of bin sizes (further details are given in^60^). Note that both $S_{m}^{*}$ and ${\mathbb {S}}_{m}$ are dimensionless. It is assumed also that in cross-sectional measurements of cross-sectional grain area and cross-sectional grain perimeter correspond to grain volume and grain area of 3D theory, respectively. So, in formula (4) $p_{i}$ are probabilities of observing certain values of cross-sectional grain area.

**Figure 1:**
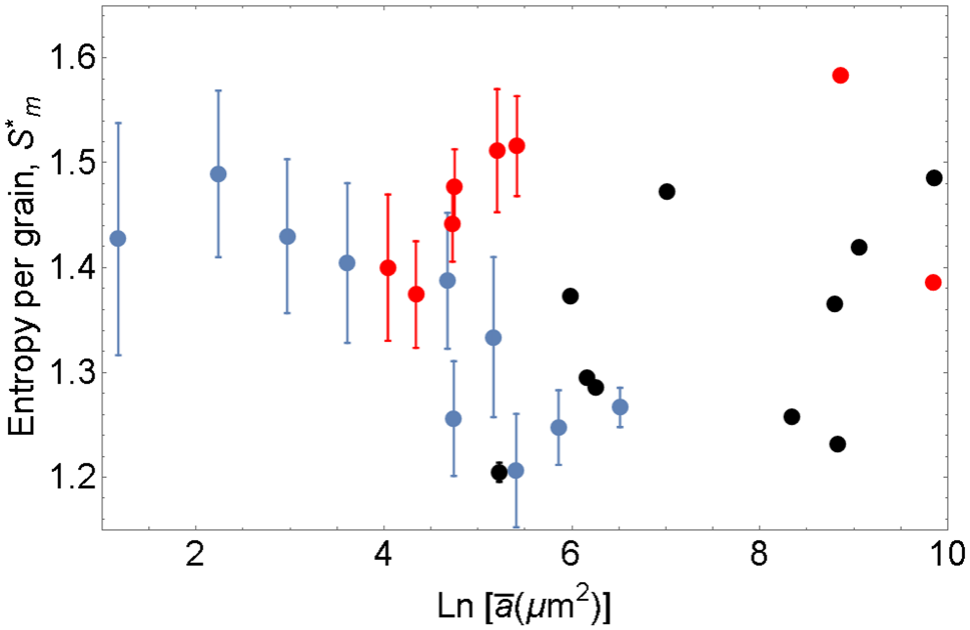
Evolution of entropy per grain ${ S}_{m}^{*}$ as a function of logarithm of mean area ${ {\bar{a}}}$ (${ \mu m}^{2}$ ). The black and red dots correspond to commercially pure nickel and aluminum alloy Al5083F^58^, respectively. Blue dots show the values computed from the data by Bhattacharya et al.^59^ for magnesium alloy AZ31bMg. Error bars are also shown. For larger grain sizes, the error bars are smaller than the displayed points.

**Figure 2:**
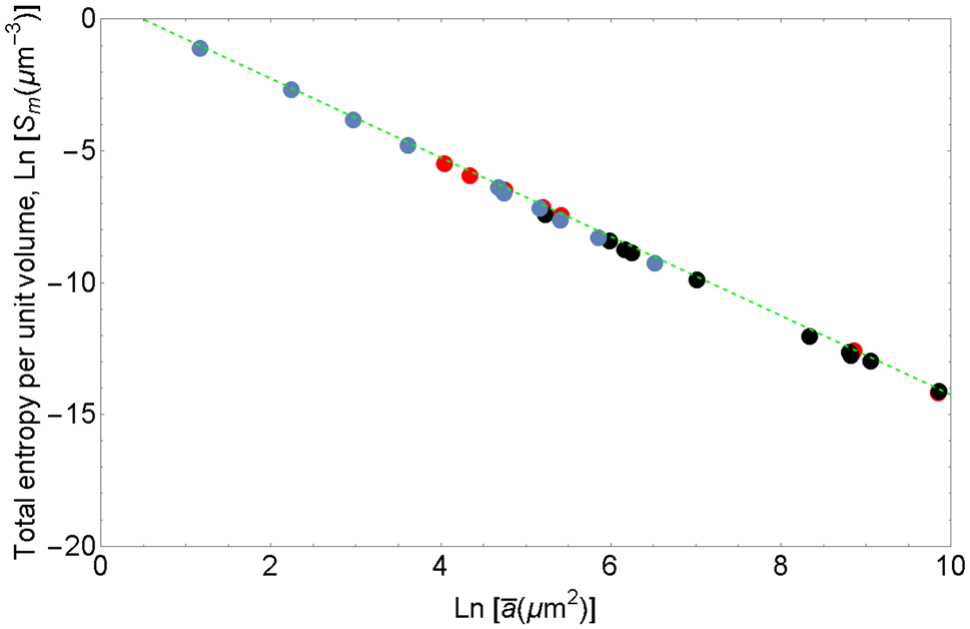
Dependence of logarithm of entropy per unit volume ${ S}_{m}$ on logarithm of mean cross-sectional grain area ${ {\bar{a}}}$ . ${ S}_{m}$ and ${ {\bar{a}}}$ are measured in ${ \mu m}^{-3}$ and ${ \mu m} ^{2}$, respectively. The black and red dots correspond to commercially pure nickel and aluminum alloy Al5083F^58^, respectively. Blue dots show the values computed from the data by Bhattacharya et al.^59^ for magnesium alloy AZ31bMg.

According to (1), the evolution of total entropy ${\mathbb {S}}_{m}$ is determined by the competetion of the decay rate of the number of grains and the rate of increase of $S_{m}^{*}$. In the analytical study^27^, grains disappear at a faster rate than the growth rate of $S_{m}^{*}$, resulting in the decay of total entropy ${\mathbb {S}}_{m}$. The experimental values of $S_{m}^{*}$ are presented in Fig. 1. It appears that $S_{m}^{*}$ does not exhibit a certain trend fluctuating slightly over the average value of 1.4. Thus, the decay of number of the grains *N* yields the decay of total entropy ${\mathbb {S}}_{m}$. The evolution of entropy per unit volume $S_{m}={\mathbb {S}}_{m}/\left| V\right|$ in grain growth is shown in Fig. 2.

Most likely, small variations of $S_{m}^{*}$ are due to the fact that all samples tested have the initial grain size distribution which is very close to self-similar distribution, and the evolution proceeds along the self-similar path.

### Entropy degeneration

**Figure 3:**
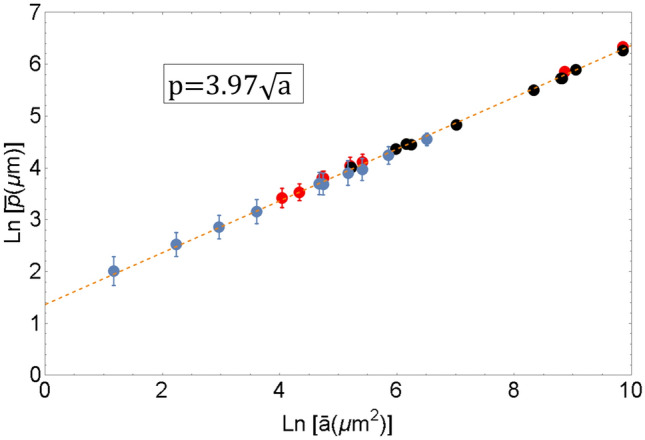
Relationship between logarithm of mean cross-sectional grain area ${ {\bar{a}}}$ and logarithm of mean cross-sectional grain perimeter ${ {\bar{p}}}$ . The black and red dots correspond to commercially pure nickel and aluminum alloy Al5083F^58^, respectively. Blue dots show the values computed from the data by Bhattacharya et al.^59^ for magnesium alloy AZ31bMg.

In general, $$S_{m}$$ is expected to be a function of energy per unit volume and grain size. For definiteness, we take as a characteristic of grain size the average grain volume *v*. Since energy per unit volume can be assumed to be proportional to average grain 3D surface area *a*, entropy per unit volume $$S_{m}$$ can be considered a function of *a* and *v*, $$S_{m}=S_{m}(a$$,*v*). Presumably, there is a link between *a* and *v* and cross-sectional characteristics of grain geometry, $${\bar{a}}$$ and $${\bar{p}}$$, which allows one to consider $$S_{m}$$ as a function of $${\bar{a}}$$, $${\bar{p}}$$. Area and perimeter are independent geometric parameters of grain cross-sections, and making measurements of $${\bar{a}}$$, $${\bar{p}}$$ and $$S_{m}$$ we expected to get a set of points in $$({\bar{a}}$$, $${\bar{p}}$$, $$S_{m})$$-space, which would yield the equation of state $$S_{m}=S_{m}({\bar{p}},{\bar{a}})$$. Surprisingly, for all microstructures at all temperatures considered the points collapse on a line shown in Fig. 2 indicating an independence of $$S_{m}$$ on $${\bar{a}}$$. The origin of such degeneration of the equation of state for $$S_{m}$$ turns out to be the existence of universal relation between $${\bar{a}}$$ and $${\bar{p}}$$. It is shown in Fig. 3. Emphasize that the points in this figure correspond to annealed microstructures obtained in a wide range of annealing times (1 min–7 days) and annealing temperatures $$(300-1100^{\circ }C)$$.

The relation between mean cross-sectional grain perimeter and mean cross-sectional grain area can be written as

5 $$\begin{aligned} {\bar{p}}=(3.97\pm 0.04)\sqrt{{\bar{a}}}. \end{aligned}$$

**Figure 4:**
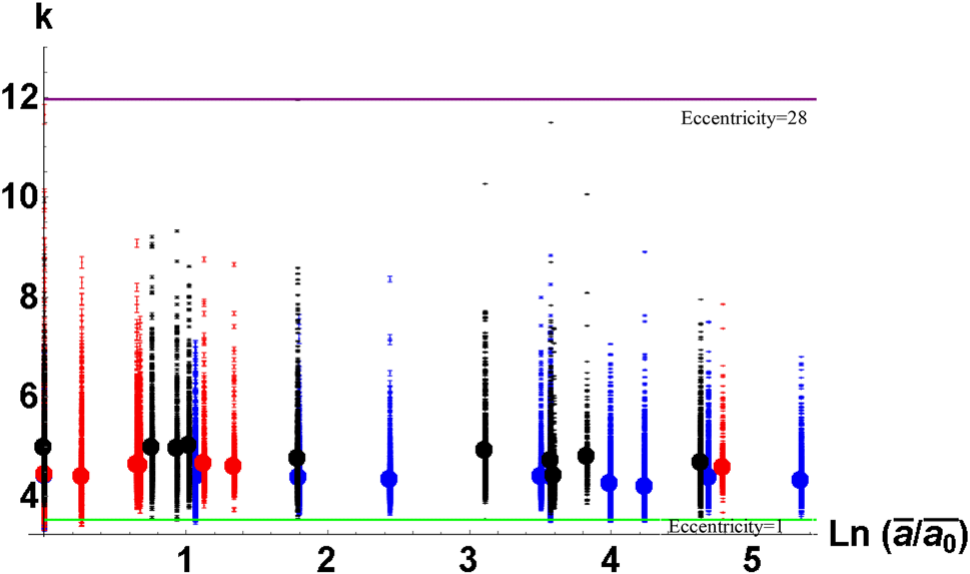
Experimental values of form factor *k* for various stages of grain growth shown in terms of mean area ${ {\bar{a}}}$ normalized by as-received sample’s mean area ${ {\bar{a}} }_{{ 0}}$. The black and red dots correspond to commercially pure nickel and aluminum alloy Al5083F^58^ respectively. Blue dots show the values computed from the data by Bhattacharya et al.^59^ for magnesium alloy AZ31bMg. Two horizontal lines show the values of eccentricity for circular cross-section (green line, ${ k=3.54}$, eccentricity 1) and grains with maximum observed eccentricity (purple line, ${ k=12}$, eccentricity 28) (Some points in Fig. 4 go below the line of unit eccentricity apparently contradicting the isoperimetric inequality: for area *a* and perimeter *p* of any 2 dimensional planar region, $4\pi a\le p^{2}$. This is due to the experimental errors which originate from errors in measurement of area and perimeter of very small grains. (See supplementary material for more details))$^{{ 2}}$.

There was a suspicion that the universality of relation (5) was caused by a special equiaxed geometry of grain structures considered. In order to check that we measured a “form factor” which is introduced for $i^{th}$ grain as the ratios, $k_{i}=p_{i}/\sqrt{a_{i}}$, $p_{i}$ and $a_{i}$ being perimeter and area of the $i^{th}$ grain cross-section (in 2D geometry, $k_{i}^{2}$ is referred to as isoperimetric quotient^61^). The observed values of form factors $k_{i}$ are shown in Fig. 4 by dots. Thick dots correspond to averaged values of form factor ${\bar{k}}.$

Since grains do not have wiggly boundaries, parameter *k* can serve as a measure of grain eccentricity. If the grain cross-section is an ellipse with semi-axes *b* and *c*, $c\ge b$, then

6 $$\begin{aligned} k=4\sqrt{\frac{e}{\pi }}E\left( \sqrt{1-\frac{1}{e^{2}}}\right) \end{aligned}$$

where $e=c/b$ is eccentricity, and *E*(*x*) is complete elliptic integral of second kind. From the measured values of $k_{i}$, one can find the corresponding eccentricity from formula (6). Two horizontal lines in Fig. 4 correspond to eccentricity equal to 1 (circle, E(0) =$\pi /2,k=2\sqrt{\pi }=3.54$) and the maximum measured value of $k=12$ corresponding to eccentricity 28. Figure 4 shows that the grain shapes vary quite noticably in the data presented in Fig. 3.

### Constitutive equations

In metallurgy, the mean grain size *R* is usually determined by measuring the number of grains *N* in a given volume *V* . Then *R* is defined as $(3V/4\pi N)^{\frac{1}{3}}$ or, in terms of average grain volume *v*, $R=(3v/4\pi )^{\frac{1}{3}}$; *v* and *R* are two interchangeable characteristics of grain size. Energy of the grain structure is proportional to average grain areas. In order to determine the dependence of energy on grain size, one has to find a link between average 3D grain area *a* and average grain volume *v*. Figure 3 suggests that there might be a relationship similar to (5),

7 $$\begin{aligned} v=\alpha a^{\frac{3}{2}}. \end{aligned}$$

As for cross-sectional geometry, 3D parameters of grain structure *a* and *v* are statistically independent, and the very fact that formula (7) holds true needs an experimental verification. No experimental results supporting the validity of (7) seem to exist, though there are various assumptions on the character of randomness of grain topology^62–69^. Our estimation^60^ of $\alpha$ is $\alpha \sim 0.1$.

If relation (7) holds true indeed, then entropy degenerates, and $S_{m}$ becomes a function of either *a* or *v*. Let us take for definiteness $S_{m}=S_{m}(v)$ and assume that 3D and 2D values of $S_{m}^{*}$ are close. Then setting $S_{m}^{*}=1.4$, we get,

8 $$\begin{aligned} S_{m}=1.4v^{-1}, \end{aligned}$$

or, in terms of *a*

$$\begin{aligned} S_{m}=1.4\alpha ^{-1}a^{-3/2}. \end{aligned}$$

The constitutive relation $S_{m}=const/v$ can be derived from dimension reasoning. If $S_{m}$ is a function of *a* and *v*, then it follows from dimension theory^50^ that

$$\begin{aligned} S_{m}=\frac{1}{v}\Phi \left( \frac{a^{3/2}}{v}\right) . \end{aligned}$$

where $\Phi$ is some function of the dimensionless variable $a^{3/2}/v$. If relation (7) holds true, then function $\Phi$ is a constant and we obtain $S_{m}=const/v.$ In general, when the dependence of grain growth on other material parameters is studied, like density of precipitates, dislocation density, texture characteristics, etc., the machinery of similarity, incomplete self-similarity, and intermediate asymptotics is expected to be quite useful as was demonstrated in other branches of materials science^51–53^. Grain boundary energy per unit volume, $U_{m}=E_{m}/V$, is

9 $$\begin{aligned} U_{m}=\frac{\gamma a}{v}=\frac{\gamma }{\alpha \sqrt{a}}, \end{aligned}$$

$\gamma$ being grain boundary energy per unit area. From (8) and (9) we get the equation of state

10 $$\begin{aligned} U_{m}=\beta S_{m}^{1/3}. \end{aligned}$$

where the parameter $\beta$ is $\gamma (1.4\alpha ^{2})^{-1/3}$. Temperature of microstructure is introduced by the usual thermodynamic relation,

11 $$\begin{aligned} T_{m}=\frac{dU_{m}}{dS_{m}}. \end{aligned}$$

The dependence of microstructure energy on $T_{m}$ is

12 $$\begin{aligned} U_{m}=c\frac{\gamma ^{3/2}}{T_{m}^{1/2}} \end{aligned}$$

where *c* is a numerical constant, $c=(\sqrt{3\cdot 1.4}\alpha )^{-1}$. According to our estimate of $\alpha$, $c\sim 4.9.$

Microstructure temperature can be expressed in terms of grain boundary energy and average grain boundary area. From (11) and (12),

13 $$\begin{aligned} T_{m}=(\alpha c)^{2}\gamma a. \end{aligned}$$

This means that up to a numerical factor microstructure temperature is equal to the average grain energy. As in other subjects of microstructure thermodynamics, $T_{m}>>T$. For grain structures, $T_{m}$ is several orders of magnitude higher than *T*. For example, for Al^56^ with $\gamma =0.1J/m^{2},$ and average grain size $R=10\mu m,$ using that $(\alpha c)^{2}\simeq 0.25$, we get for the ratio of $T_{m}$ to melting temperature $T_{melt\text { }}$(933 K) the value $T_{m}/T_{melt}=6\times 10^{8}.$

## Concluding remarks

Classical equilibrium thermodynamics is a “coarse-grained” description of ergodic Hamiltonian systems as was first understood by Gibbs and Boltzmann. Far from equilibrium, the systems exhibit two types of behavior. The systems can either remember well their “Hamiltonian origin”, or forget it becoming truly dissipative and retaining the memory of the “Hamiltonian origin” only in the structure of energy and dissipation. The examples of first kind are gases, fluids, amorphous solids. The second type of behavior is typical for dynamics of mesoscopic defects of crystals, like dislocation ensembles or grain boundaries. The basic characteristic of the second type cases is the possibility to describe them by evolutionary dissipative equations. Navier-Stokes equations governing turbulent motions of fluids is an example of dissipative systems which need a coarse graining though on macroscopic rather than mesoscopic level. The major peculiarity of thermodynamic (coarse-grained) theory of dissipative systems is a huge diversity of possible phase flow geometries. This is to the contrary to ergodic Hamiltonian systems which are all alike: phase space is split in a family of energy surfaces, and phase flow is ergodic on each energy surface. It is this similarity and the Hamiltonian structure of the microworld dynamics that make equilibrium thermodynamics so universal. Thermodynamics of dissipative systems is expected to be quite more system-specific.

The first consistent generalization of the notion of entropy for non-equilibrium systems was given by Leontovich^54^. His idea was to introduce some external actions on the system to force the non-equilibrium states to become equilibrium. Then entropy of such states (non-equilibrium entropy) is a function of usual thermodynamic parameters and the parameters of the external action. This approach works for many non-equilibrium physical systems^55^.

What is entropy of dissipative systems is not clear. At the moment some insight can be gained by studying particular cases. The only general concept is that entropy must decay if it is associated with the phase volume and the system is isolated. Emphasize that entropy could be of interest from the perspective of a macroscopic theory only if entropy enters the coarse-grained (averaged) constitutive equations. From this perspective, it is not clear whether the parameter $S_{m}$ studied in this paper is essential because a meaningful coarse-grained thermodynamic theory of grain growth does not exist at the moment. Besides, the most simple set of thermodynamic parameters considered, *a* and *v*, turns out to be degenerated, and grain growth dynamical equation can be formulated without using $S_{m}$. Whether entropy considered is essential or not will be clear when more physical effects are taken into account: the grain boundary interactions with precipitates and dislocations, influence of texture distribution, plastic deformation, etc. The experimental data reported supports the assertion that entropy of microstructure decays in the process of grain growth.

It is noteworthy that for one-parametric models like the one specified by (10) entropy decay is a consequence of the first and the second laws of thermodynamics. Indeed, according to the first law of thermodynamics, in an isolated system total energy *E* is conserved. In grain growth, *E* is a sum of energy of atomic motion, $E_{th}$, and energy of grain boundaries, $E_{m}$. The first law of thermodynamics reads:

14 $$\begin{aligned} \frac{dE}{dt}=\frac{dE_{th}}{dt}+\frac{dE_{m}}{dt}=0. \end{aligned}$$

According to second law of thermodynamics, thermodynamic entropy ${\mathbb {S}}_{th}$ increases,

15 $$\begin{aligned} \frac{dE_{th}}{dt}=T\frac{d{\mathbb {S}}_{th}}{dt}>0. \end{aligned}$$

In (15)$\ T$ is the absolute temperature which is defined as $T=dE_{th}/d{\mathbb {S}}_{th}$. Assuming that microstructure temperature $T_{m}$ is positive,

16 $$\begin{aligned} T_{m}=\frac{dE_{m}}{d{\mathbb {S}}_{m}}=\frac{dU_{m}}{dS_{m}}>0, \end{aligned}$$

We obtain from (14) that microstructure entropy decays,

17 $$\begin{aligned} \frac{d{\mathbb {S}}_{m}}{dt}=-\frac{T}{T_{m}}\frac{dS_{th}}{dt}<0. \end{aligned}$$

Note that microstructure entropy decay would not follow from the first and second laws of thermodynamics and would be an independent statement, if microstructure energy $E_{m}$ was a function of both arguments, *v* and ${\mathbb {S}}_{m}$.

The equation (12) and (13) allow us to check the consistency of the assumption that grain growths in adiabatically isolated setting and in a thermal bath are practically the same. Indeed, from the conservation of energy in adiabatically isolated system and equation (12), we have

18 $$\begin{aligned} c_{V}T+c\frac{\gamma ^{3/2}}{T_{m}^{1/2}}=c_{V}\mathring{T}+c\frac{\gamma ^{3/2}}{\mathring{T}_{m}^{1/2}} \end{aligned}$$

where $c_{V}$ is heat capacity per unit volume, $\mathring{T},$ $\mathring{T}_{m}$ are the initial values of absolute temperature and microstructure temperature. Knowing the initial grain size and initial temperature, we can find from (18) temperature of the specimen after a certain grain size increase. For example, for Al^56,57^ with $\gamma =0.1J/m^{2},$ $c_{V}=2.4\times 10^{6}J/m^{3}K,$ initial average grain size $\mathring{R}=10\mu m,$for a typical temperature setting $\mathring{T}=700K,$ after an order of magnitude of grain size increase, we have using (13) $\mathring{T}_{m}=6\times 10^{11}K,T_{m}=6\times 10^{13}K$. Then from equation (18) temperature increase is $T-\mathring{T}=10^{-3}K$. This is within the experimental errors and grain boundary mobility remains practically the same. Thus, the grain structure evolution in adiabatic and isothermal settings can hardly be distinguished. Of course, the origin of that is the huge difference between the values of energy of atomic motion and energy of grain boundaries. In our example, at the start of grain growth, $c_{V} \mathring{T}=1.7\times 10^{9}J/m^{3}$, while the initial value of microstructure energy density is $U_{m}=2821J/m^{3}.$

The degeneration of constitutive equations which we observed is likely due to the fact that in all the samples tested grain growth followed a self-similar path. In this regard, it would be interesting to study grain growth in materials with bimodal or trimodal initial grain size distribution alongwith another open question which is to get an experimental verification of relation (7).

## Supplementary information

Supplementary information.
