# Supplementary material for: Entropy decay during grain growth
Source: Sci Rep. 2020 Jul 17;10:11912. doi: 10.1038/s41598-020-68569-z (PMC7367831; doi:10.1038/s41598-020-68569-z)
Supplement: Supplementary file 1 — Supplementary information. [file 41598_2020_68569_MOESM1_ESM.pdf]

Supplementary materials for

ENTROPY DECAY DURING GRAIN GROWTH

Pawan Vedanti, Xin Wu and Victor Berdichevsky

Wayne State University

(Dated: Dec 2019)

Abstract

In this text we describe in more detail the experiments carried out in this work. Materials section describes experiment performed on each material studied, Methods section focuses on data collection and analysis and Estimation of α section suggests a way to evaluate the parameter α in the constitutive equation. We also provide pictures of microstructures and plots for probability distribution of grain sizes illustrating the self-similarity during grain growth experiment.

I. MATERIALS

A. Nickel microstructure analysis

The nickel samples were cut 10-12mm each from commercially pure 0.25" rod (from McMaster Carr). The annealing of samples was done in closed furnace for all the samples for different temperatures and times. The sample was put in the furnace roughly 100°C before it reached the required annealing temperature. Once the annealing time was complete, sample was removed and air cooled to bring it back to room temperature. Each sample was then hot mounted using epoxy. The mounted samples were then prepared for EBSD by hand polishing. The following SiC grit papers were used: 180, 320, 600, 1200. Cloth polishing was done with 5 different sizes of diamond paste. The final polishing was done using colloidal silica solution on a silk cloth and the time required for each sample was between 30 mins to 1 hour for this step. Post-polishing, the samples were cleaned using a ultrasonic cleaner to make sure no residual dust particles exist.

EBSD of each sample was done as close to the center as possible in order to avoid the edges and free surface. The instrument used for EBSD is JSM 7600 FE SEM. The voltage of SEM was set at 20kV for all the scans. Multiple scans were done on different areas of the sample so as to make sure that we have atleast 300-350 grains for each of the conditions. The step size varies from 2 um for as received to 15 um for the samples heated at high temperature for longer time. After getting the EBSD scans through OIM data collection software, they were analyzed and all points with CI < 0.1 were removed. Grain dilation method was used for getting the final cleaned image for each scan. All the grains were then hand-traced using Image-J software to get the statistical information about the area and perimeter. The error in the measurement is calculated from the minimum area and perimeter measureable by the software which is usually 1-4 pixels of the image. Example images are shown in fig. $S1$.

B. Magnesium microstructure analysis

The detailed method of getting the magnesium microstructure images has been explained in [51]. Optical microscope image of surface of annealed samples after etching are shown below in fig. $S2$. The grain boundaries which are visible in the images were hand-traced and

the area near the edges was avoided to gather data from the grains which are completely captured in the image.

C. Aluminum microstructure analysis

Al 5083F is a fine-grained aluminum alloy developed by Alcoa for superplastic forming and the grain growth data for this material was provided by Dr. Huibin Wu[50]. The post polishing EBSD microstructure images (example shown in fig. $S3$) were also processed the same way as the nickel samples (by hand tracing).

II. METHODS

A. Microstructure cross-section topology

Once the microstructure image is obtained, grain boundaries were hand traced. All the lighter components of the image were thresholded in order to highlight the skeleton of traced grain boundary network. Fig. $S4$ shows examples of images before and after grain boundary tracing. Sources of error in measurement of cross-sectional area and perimeter: There are systematic errors which inherently exist when carrying out the analysis of the traced grain boundary images. Image-J has an adjustable parameter (a_m) specifying minimum measureable area. Range of a_m is 0.25 μm^2 to 10 μm^2 depending on the value of mean cross-sectional area of the microstructure. As a_m is specified manually, this leads to the software ignoring grains smaller than a_m. This leads to overestimation of mean 2D characteristics of grain structure. Tracing of grain boundaries was done with a brush of fixed width (2 pixels) which is the source of error in perimeter measurement. p_m, minimum measureable perimeter will be of the order of width of traced grain boundaries. Range of p_m is 0.5 μm to 2 μm. Error in measurement of cross-sectional area and perimeter of grains propogates further in the calculation of entropy per grain S_m^* and microstructure entropy per unit volume S_m.

$$Err_a = \frac{a_m}{\bar{a}}, Err_p = \frac{p_m}{\bar{p}}. \tag{1}$$

where Err_a and Err_p are measurement errors in cross-section area and perimeter. The overall effect of these errors is significant on the microstructure images with smaller average

grain size. We get less than 2% error as grain growth proceeds.

B. Calculation of 2D characteristics

The cross-sectional area and perimeter of each grain are known. This allows one to calculate the mean value of the 2D parameters using formula shown below.

$$\bar{a} = \frac{a_1 + a_2 + ... + a_{N-1} + a_N}{N}, \ \bar{p} = \frac{p_1 + p_2 + ... + p_{N-1} + p_N}{N}. \tag{2}$$

where $\bar{a}$ and $\bar{p}$ are mean cross-sectional area and perimeter, respectively, a_i is the 2D cross-sectional area of ith grain, p_i is the 2D cross-sectional perimeter of ith grain and N is the total number of grains measured in the section. Additional characteristics of the microstructure can be determined from the measurement of cross-sectional area and perimeter of each grain. A dimensionless form factor, k_i has been introduced for i^{th} grain. Mean value of measured k_i for a given microstructure denoted by K is also calculated (shown in fig. 4 as large dots). Another parameter $\bar{k}$ is calculated shown in fig. 3 using the following relation

$$\bar{k} = \frac{\bar{p}}{\sqrt{\bar{a}}}. \tag{3}$$

where $\bar{a}$ and $\bar{p}$ are known mean cross-sectional area and perimeter, respectively.

C. Grain size distribution

Volume of the grain is estimated based on the assumption that grains are spherical. This may not always be the case but it has been proven to be a convenient approximation. The equivalent circle diameter d_a and equivalent sphere diameter d_s are calculated using equation shown.

$$d_a = \sqrt{\frac{4a_i}{\pi}}, d_a = \frac{\pi}{4}d_s. \tag{4}$$

The volume of individual grain v_i will then be given by

$$v_i = \frac{\pi}{6}d_s^3. \tag{5}$$

The next step of the analysis is to get the grain size (normalized volume) distribution of the microstructure. Average volume of the microstructure $\bar{v}$ is calculated in the same way

as cross-sectional area and perimeter shown in (2). Each grain volume, v_i is normalized by average volume giving one dimensionless number $(v_i/\bar{v})$ to characterize a grain. From (4) and (5), one can observe that normalized volume and normalized area are linked as

$$\frac{v_i}{\bar{v}} = (\frac{a_i}{\bar{a}})^{\frac{3}{2}} \tag{6}$$

Normalized volume of all the grains is then divided into bins to count the total number of grains within that bin. The probability of finding a grain in a particular bin is the ratio of number of grains in the said bin to the total number of grains. After getting grain size distribution, entropy per grain is calculated. Fig. $S5$ shows example of grain size distribution for different metals and their respective stages of annealing. The initial and final volume distribution of all the chosen materials is far from self similar as the microstucture is evolving towards a steady-state.

D. Calculation of entropy per grain, S^*_m

Entropy per grain for a given microstructure is calculated from the definition of S^*_m as given by (4). The value of entropy per grain S^*_m is dependent on selected bin size. The reasoning is that the value of probability of finding a particular grain size is dependent on the bin size chosen hence affecting the final size distribution (example shown in fig. $S6$a) qualitatively. Fig. $S6$b shows how S^*_m depends on bin size. To make S^*_m invariant to bin size, the following method is applied: Entropy per grain S^*_m for each individual sample is found out by taking an average over a range of bin sizes. The bins chosen for this study are 0.25, 0.4, 0.5, 0.6 and 0.75. This leads to a simplification in calculation of S^*_m.

$$S^*_m = -\sum_i \frac{n_i}{N} \ln \frac{n_i}{N}. \tag{7}$$

Here n_i is the count of grains in i^{th} bin and N is the total number of grains considered. Microstructure entropy per unit volume is estimated as,

$$S_m = S^*_m/\bar{v}. \tag{8}$$

III. EVALUATION OF α

Usually grain size is found by linear intercept method in most of the grain measurement experiments. The linear intercept yields the ratio of volume to surface area.

$$\frac{N_A}{L} = \frac{2}{\pi} L_A, \; \frac{N_A}{L} = \frac{1}{2} S_V. \tag{9}$$

where P_L is the count of intersections per unit length of line, N_A is the number of intersections, L is the total length of the line intercept drawn, L_A is the ratio of 2D cross-sectional perimeter to area and S_V is ratio of 3D boundary surface area per unit volume. From (9),

$$S_V = \frac{4}{\pi} L_A. \tag{10}$$

In terms of our paper, eq. (10) can be re-written as

$$\frac{a}{v} = \frac{4}{\pi} \frac{\bar{p}}{\bar{a}}. \tag{11}$$

where a, v are 3D average grain area and volume respectively, and $\bar{p}$, $\bar{a}$ are 2D cross-sectional mean perimeter and area respectively. Introducing the definition of "form factor" into eq. (11), α in (7) can be found out in terms of the known quantities as

$$\alpha = \frac{0.7}{\bar{k}^{\frac{3}{2}}} \frac{\bar{a}^{\frac{3}{4}}}{v^{\frac{1}{2}}}. \tag{12}$$

If $\bar{k} = 4$ is taken as a constant from the experimental data from fig. 3 and fig. 4 of the main text. If the ratio of 2D cross-sectional grain area and 3D volume, $\bar{a}^{\frac{3}{4}}/v^{\frac{1}{2}}$ is considered to be unity, then one gets $\alpha \sim 0.1$ in (7). For reference, a regular sphere and a regular cube have $\alpha = 0.095$ and $\alpha = 0.068$, respectively. Emphasize, that the relation (10) used for this estimation is based on the assumption which can be interpreted as the ergodicity of space tesselation.

IV. MICROSTRUCTURE FIGURES

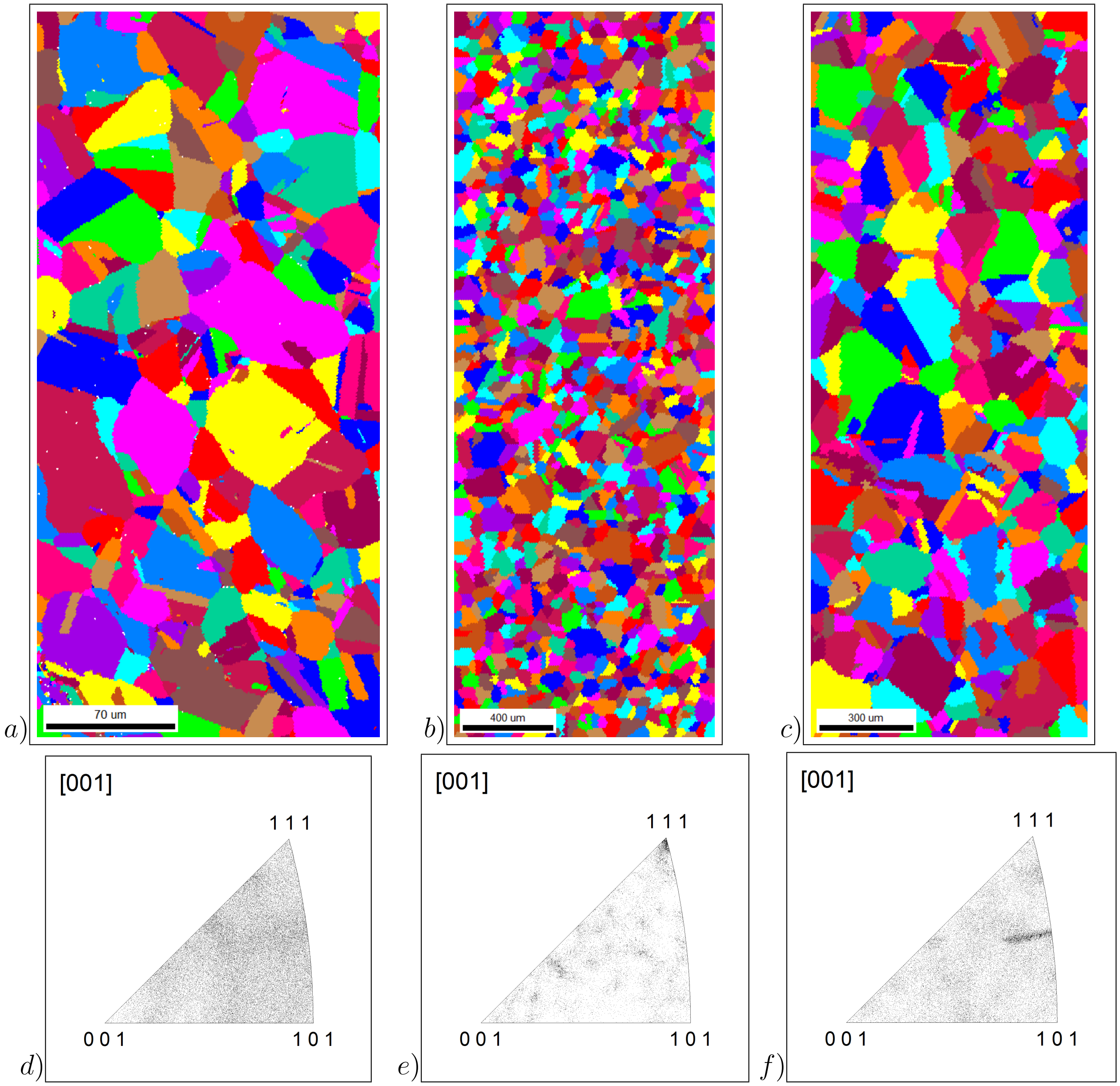

Fig. $S1$. a), b) and c) are EBSD images of as-rec, 850C 0.5h, 1000C 3h nickel samples, respectively whereas d), e) and f) are the corresponding orientation image maps for the same samples.

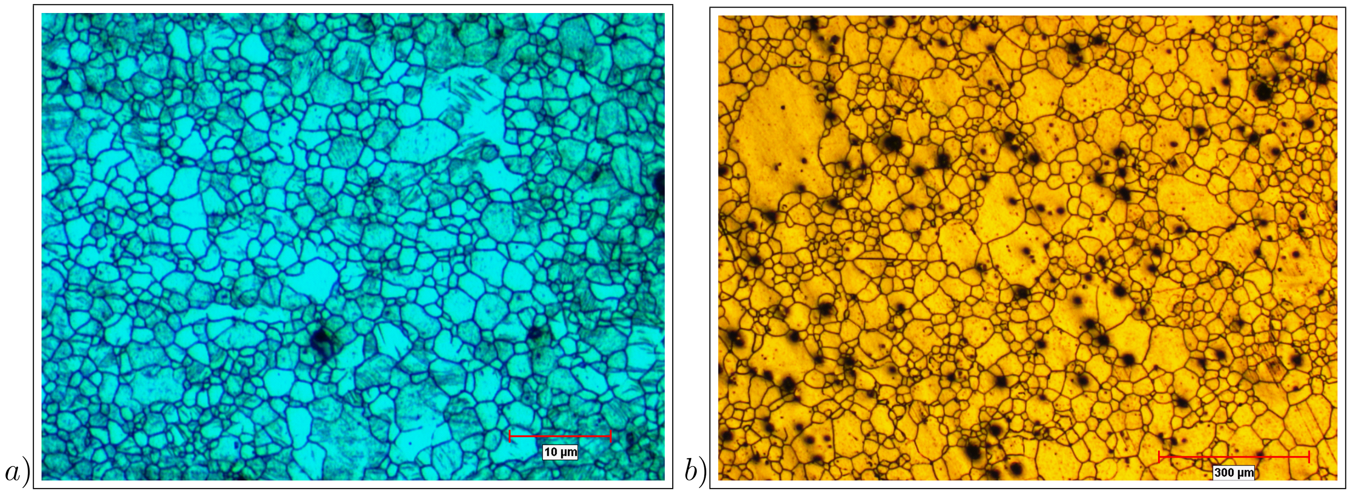

Fig. $S2$. a) and b) shows optical microscope images for AZ31bMg after etching of as-rec and annealed at 450°C for 22 hours.

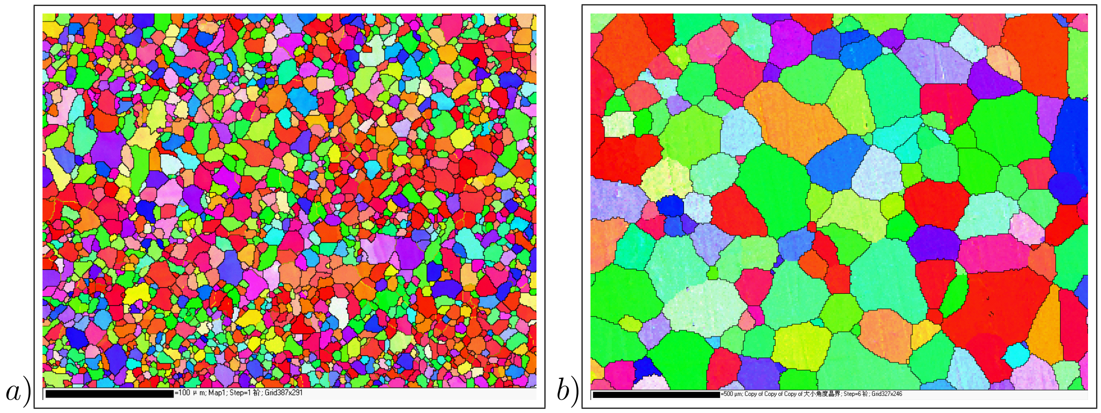

Fig. $S3$. a) and b) shows EBSD images for Al5083F of as-rec and annealed at 600°C for 5 hours.

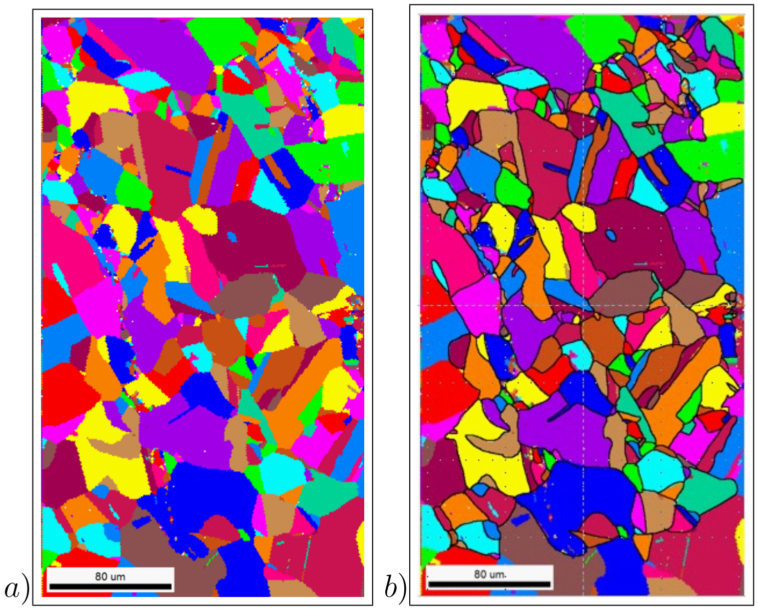

Fig. $S4$. As-received Ni sample a) before tracing b) highlighted grain boundaries after tracing

V. SELF-SIMILARITY OF GRAIN GROWTH

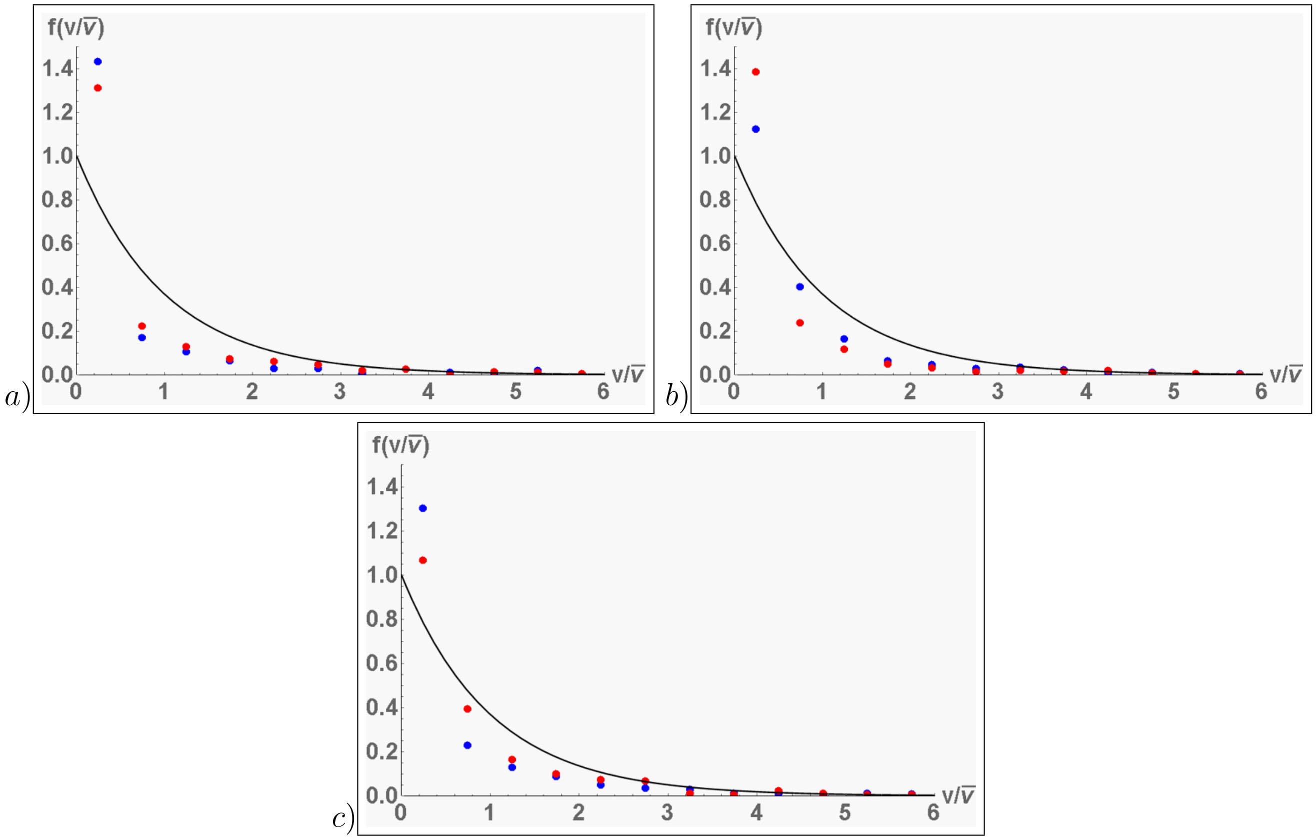

Fig. $S5$ a) Grain size (normalized volume) distribution for as-rec (blue dots) and sample annealed at $1000°C$ for 180 min (red dots) nickel samples, b) Grain size (normalized volume) distribution for as-rec (blue dots) and sample annealed at $450°C$ for 22 hr (red dots) AZ31bMg samples, c) Grain size (normalized volume) probability distribution for as-rec (blue dots) and sample annealed at $600°C$ for 1 hr (red dots) Al5083F samples. The black curve is exponential distribution which corresponds to self-similar grain growth[49]. The bin size used here is 0.5.

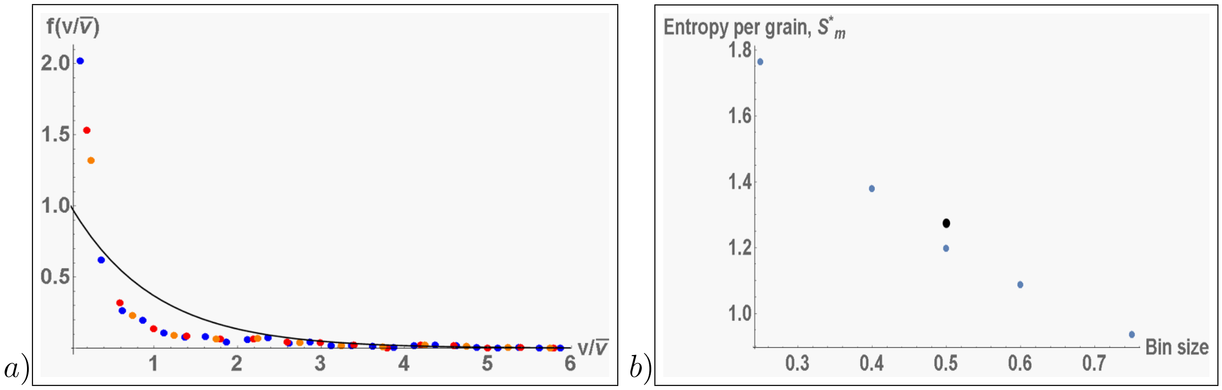

Fig $S6$ a)Volume probability distribution of annealed (Temp.- $850°C$,time - 240 min) nickel sample. S^*_m values are 1.8, 1.4 and 1.2 for bin sizes 0.25 (blue dots), 0.4 (red dots) and 0.5 (orange dots), respectively. b) Dependence of S^*_m on bin sizes for the same sample. The black dot is average value of S^*_m over the selected range of bin sizes.

VI. TABLES

Table $S1$. Temperatures and times of annealing for Ni samples

Temperature ($°C$)	Time (min)		
850	5	30	240
1000	5	30	180
1100	5	30	90

Table $S2$. Temperatures and times of annealing for AZ31bMg samples

Temperature ($°C$)	Time (min)		
300	4	4320	10080
400	4	60	1080
450	1	4	1320

Table $S3$. Temperatures and times of annealing for Al5083F samples

Temperature ($°C$)	Time (min)	
450	-	60
500	60	180
550	60	180
600	60	300
